# Triglycerides are related to left ventricular mass in hypertensive patients independently of other cardiometabolic risk factors: the effect of gender

**DOI:** 10.1038/s41598-020-70237-1

**Published:** 2020-08-06

**Authors:** Panagiota Pietri, George Georgiopoulos, Dimitrios Tsiachris, Athanasios Kordalis, Charalambos Vlachopoulos, Gregory Vyssoulis, Christodoulos Stefanadis

**Affiliations:** 1 Athens Heart Center, Athens Medical Center, Kythnou 15, 15231 Athens, Greece; 2 1st Cardiology Department, Athens Medical School, University of Athens, Athens, Greece

**Keywords:** Biomarkers, Cardiology, Risk factors

## Abstract

Given the inconsistent results on the prognostic significance of triglycerides (TGs), the purpose of the present study was to investigate the association of plasma TGs with left ventricular mass (LVM) in hypertensive patients. We studied 760 never treated, non diabetic, hypertensive patients. Τransthoracic echocardiography was performed and LVMI was calculated according to the Devereux formula, adjusted to body surface area. Triglycerides were associated with LVMI after adjustment for age, gender, systolic blood pressure (SBP), smoking and fasting glucose (b = 0.08, p = 0.009). This relationship remained significant even after adjustment for BMI, LDL-C and ApoB/ApoA1 ratio (b = 0.07, p = 0.04). Gender-stratified analysis indicated that TGs were related to LVMI in men (p = 0.001) but not in women (p = NS). In addition, TGs were related with LV hypertrophy (LVH) in men, increasing the odds by 7% to present LVMI over 115 g/m^2^ (OR = 1.07 per 10 mg/dl increase in TGs, p = 0.01). In conclusion, TGs are associated with LVMI in hypertensive patients, independently of other risk factors, including LDL-C. Given the prognostic significance of LVH, it might be suggested that TGs may serve as a useful marker for indentifying hypertensive patients at high risk. The gender discrepancy may suggest a possible gender-specific modulatory effect of TGs on LV structure.

## Introduction

Plasma total cholesterol and low-density lipoprotein cholesterol (LDL-C) are independent predictors of cardiovascular disease and their measurement is strongly recommended for risk stratification. Moreover, in the recent guidelines, plasma LDL-C remains the prevailed treatment target for both primary and secondary prevention with the optimum target level to become even lower than in the previous years^1^.

Plasma triglycerides (TGs) add information on cardiovascular risk, whereas genetic data support a direct effect of TGs on cardiac disease^2,3^. Interestingly, high levels of plasma TGs may explain part of the residual cardiovascular risk in statin-treated patients, including hypertensive patients, with optimal LDL-C levels^4^. However, results concerning the cardiovascular benefit of TGs reduction are contradictory. Indeed, previous meta-analysis did not demonstrate a significant association of omega-3 fatty acid supplementation with fatal or non fatal cardiovascular events in the patients with prior coronary heart disease^5^. On the contrary, in the large, multicenter, randomized REDUCE-IT trial, lowering of plasma TGs with eicosapentaenoic acid ethyl ester, reduced the risk of ischemic events, including cardiovascular death, among patients with established cardiovascular disease who had been receiving statin therapy and had high triglyceride level^6^.

Left ventricular hypertrophy (LVH) confers an independent risk of future cardiovascular events in hypertensive patients. Data in hypertensive patients have established an adverse effect of low plasma HDL-C on left ventricular mass (LVM)^7–9^. However, the evidence on the association of plasma TGs with LVMI in hypertensive patients is limited.

Given the prognostic significance of LVH and the limited data on the role of plasma TGs in LV structure of hypertensive patients, we sought to investigate the association of LVM with plasma TGs in never treated hypertensive male and female patients.

## Results

Baseline characteristics of the study hypertensive patients are shown in Table 1. Regarding the differences in anthropometric, biochemical and hemodynamic characteristics between the two genders, the following were observed: females were slightly older than males (54 ± 13 vs 52 ± 13 year, p = 0.05) with similar body mass index (BMI) values (27 ± 5 vs 27 ± 3 kg/m^2^, p = NS). The percentage of smokers was lower in females compared to males, although with no statistical significance (29 vs 34%, p = NS). Systolic blood pressure (SBP) did not differ between the two genders (162 ± 14 vs 161 ± 14 mmHg, p = NS) but diastolic blood pressure (DBP) was slightly higher in men compared to women (104 ± 7 vs 102 ± 7 mmHg, p = 0.004). Regarding the metabolic profile, no difference in plasma fasting glucose (96 ± 12 vs 95 ± 12 mg/dl, p = NS) and LDL-C (157 ± 40 vs 156 ± 43 mg/dl, p = NS) was observed between male and female patients. However, women exhibited higher plasma HDL-C (53 ± 12 vs 45 ± 9 mg/dl, p < 0.001) and TGs levels (100 vs 82 mg/dl, p = 0.02) and lower ApoB/ApoA1 ratio (0.79 vs 0.89, p < 0.001) compared to men. Finally, LVMI was significantly increased in males compared to females (125 vs 110 g/m^2^, p < 0.001). However, the percentage of male and female patients with LVH did not differ (49.5 vs 49.7%, p = NS).

**Table 1:** Baseline characteristics of study hypertensive patients.

Age, years	52 ± 13
Males, N (%)	414 (54)
BMI, kg/m^2^	27 ± 4
Smokers, N (%)	243 (32)
SBP, mmHg	161 ± 14
DBP, mmHg	103 ± 7
MAP, mmHg	122 ± 6
Fasting glucose, mg/dl	95 ± 11
Total cholesterol, mg/dl	229 ± 44
LDL cholesterol, mg/dl	156 ± 41
HDL cholesterol, mg/dl	49 ± 11
Triglycerides, mg/dl (median)	105
ApoB/ApoA1 ratio (median)	0.83
LVMI, g/m^2^ (median)	120
LVH, N (%)	377 (49.6)

In the whole population, abnormal glucose metabolism was demonstrated in 37% of patients, defined either as impaired fasting glucose (IFG) or impaired glucose tolerance (IGT). Patients with IFG/IGT had increased LVMI compared to hypertensive patients with normal glucose metabolism (124 vs 118 g/m^2^, p < 0.001).

The bivariate correlation, in the whole population, showed that LVMI was related to age (r = 0.29, p < 0.001), gender (r = 0.31, p < 0.001), smoking (r = 0.13, p < 0.001), SBP (r = 0.36, p < 0001), mean arterial pressure (MAP) (r = 0.25, p < 0.001), BMI (r = 0.13, p < 0.001), plasma fasting glucose (r = 0.25, p < 0.001), plasma LDL-C (r = 0.12, p = 0.001), plasma HDL-C (r = − 0.21, p < 0.001), ApoB/ApoA1 ratio (r = 0.21, p < 0.01) and plasma TGs (r = 0.23, p < 0.001).

**Figure 1:**
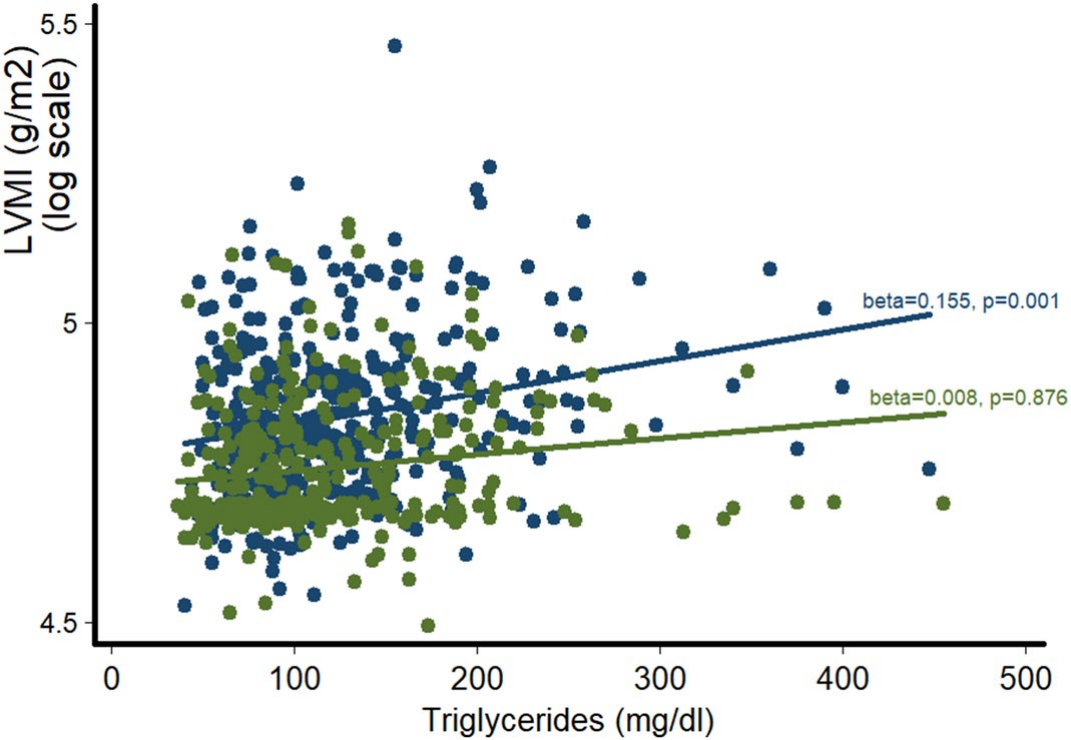
Differential association of LVMI with plasma TGs in men (blue dots and line) and women (green dots and line). Beta coefficients and p-values are derived from multiple regression analysis of (log) LVMI on TGs after controlling for age, SBP, fasting glucose and smoking.

In multiple regression analysis, in the whole population, LVMI was related to plasma TGs after controlling for age, gender, smoking, SBP, and plasma fasting glucose (b = 0.08, p = 0.009). This relationship remained significant even after adjustment for BMI, LDL-C and ApoB/ApoA1 ratio (Table 2). An interaction term of plasma TGs with gender was significant in the final multivariable model, both in continuous and dichotomous format (p = 0.009 and p = 0.03 for TGs as a continuous variable*gender and hypertriglyceridemia*gender, respectively). Stratified analysis by gender indicated that TGs were differentially associated with LVMI. In particular, TGs were related to LVMI in men (b = 0.15, p = 0.001) but not in women (p = NS) after adjustment for age, smoking, SBP and plasma fasting glucose (Fig. 1). This association was remained significant even after adjusting for additional confounders, including BMI, plasma LDL-C and ApoB/ApoA1 ratio.

**Table 2:** Multiple regression analysis of the association of (log) LVMI with (log) plasma TGs in hypertensive patients, after adjustment for age, gender, smoking, BMI, SBP, plasma fasting glucose, LDL-C and (log) ApoB/ApoA1 ratio.

	Standardized regression coefficient	p value
Age	0.19	< 0.001
Male gender	0.33	< 0.001
SBP	0.25	< 0.001
Smoking	0.11	0.001
Glucose	0.09	0.005
TGs	0.07	0.04
LDL-C	0.02	NS
ApoB/ApoA1 ratio	0.02	NS

R^2^ = 0.31.

**Figure 2:**
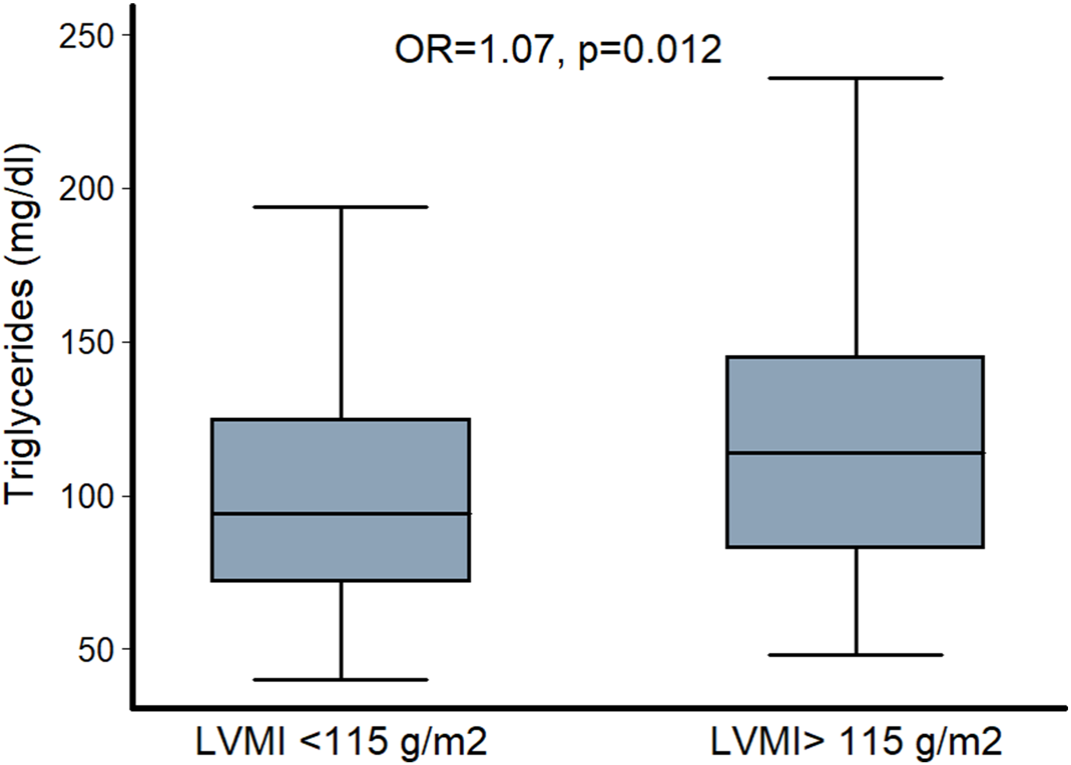
Difference in plasma TGs between hypertensive males with and without LV hypertrophy (LVMI below or above 115 g/m^2^). p-value was derived from logistic regression analysis after adjustment for age, SBP, plasma fasting glucose and smoking.

**Figure 3:**
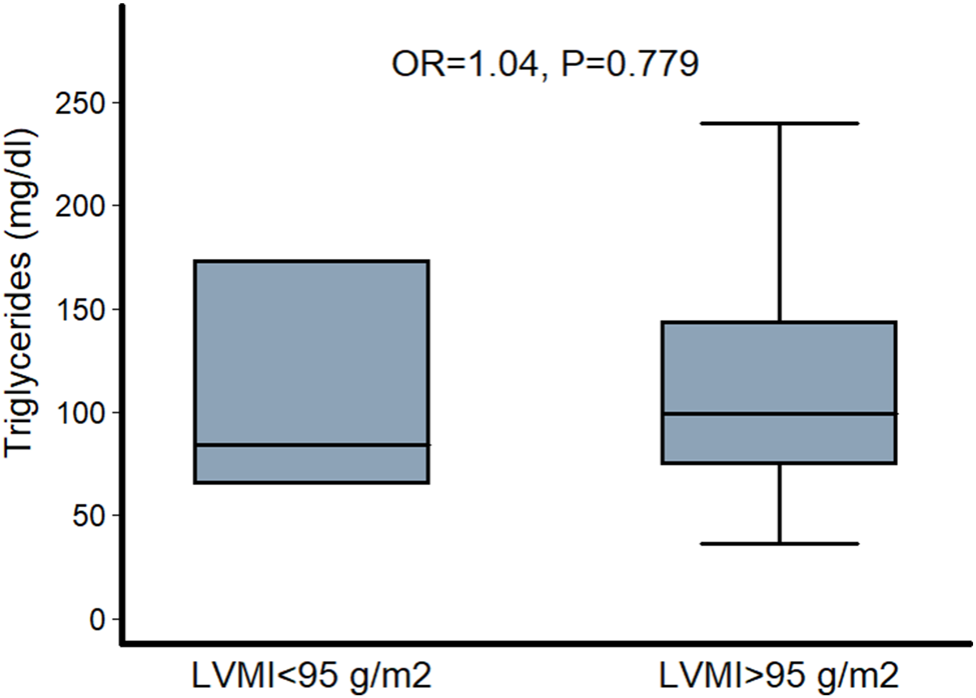
Difference in plasma TGs between hypertensive females with and without LV hypertrophy (LVMI below or above 95 g/m^2^). p-value was derived from logistic regression analysis after adjustment for age, SBP, plasma fasting glucose and smoking.

In the final step of our analysis, we identified male subjects that presented LVH and tested if plasma TGs were independently associated with LVH in this subgroup. Importantly, plasma TGs were associated with LVH (OR = 1.07 per 10 mg/dl increase in plasma TGs, 95% CIs 1.02–1.14, p = 0.01) (Fig. 2) and increased the odds by 7% to present LVH over 115 g/m^2^ after controlling for age, smoking, SBP and plasma fasting glucose. This association was not attenuated when other cardiometabolic risk factors, including BMI, plasma LDL-C and ApoB/ApoA1 ratio, were taken into account. Plasma TGs reclassified male subjects into correct categories for the presence or absence of LVH over the core model (age, smoking, SBP and fasting glucose) (continuous net reclassification index, NRI = 38.2%, p = 0.001). The incremental value of TGs over established cardiometabolic risk factors was evident even after additional adjustment for BMI, LDL-C and ApoB/ApoA1 ratio (continuous NRI = 24.1%, p = 0.04). On the contrary, plasma TGs were not associated with LVH in females (Fig. 3).

## Discussion

In the present study, we demonstrated a strong relationship of LVMI with plasma TGs in hypertensive patients, independently of other cardiometabolic risk factors including plasma LDL-C. Moreover, for the first time, a gender discrepancy considering this association was emerged, with potential pathophysiogical, clinical and prognostic implications.

The longitudinal effect of lipids on LVM was first elucidated by Sundström et al. who showed that the high intake of saturated and monounsaturated fats predict the future prevalence of LVH in the general population^10^. Limited data in hypertensive population come from small studies which have shown a relationship of low plasma HDL-C with LVM and diastolic function^7–9^. Plasma triglycerides have been associated with LVH^11^, although with inconsistent results^8^. However, in the above studies, plasma LDL-C was not included as a confounder in the linear regression analysis and the incremental value of plasma TGs was not investigated.

Our present findings support a strong, independent association of plasma TGs with LVMI in hypertensive patients, over and above plasma LDL-C. Given the prognostic significance of LVH, this finding may, at least in part, explain the residual cardiovascular risk observed in patients with optimal plasma LDL-C levels.

The pathophysiological mechanisms underlying this relationship are not fully elucidated. However, it might be argued that insulin resistance may mediate part of the association of LVMI with plasma TGs given the data on the unfavorable role of insulin resistance on LVstructure and function^12–15^. To further support this notion, the strong independent relationship of LVMI with fasting glucose in our population may imply a possible modulatory role of abnormal glucose metabolism, through the mechanism of insulin resistance. Although we recruited non-diabetic patients, a part of our population exhibited abnormal glucose metabolism defined either as IFG or IGT. This sub-population had increased LVMI compared to hypertensive patients with normal glucose metabolism. Data have shown that non-diabetic individuals with IFG and IGT have a 3 to tenfold greater probability of LVH compared to subjects with normal glucose tolerance^16^. Aortic stiffness, a major determinant of LVH may also serve as a potential mediator to the relationship of plasma TGs with LVMI given previous results demonstrating an association of plasma TGs and impaired glucose metabolism with aortic stiffness^17–20^.

From a mechanistic perspective, a more prominent underlying mechanism explaining the adverse effect of atherogenic dyslipidemia, mainly plasma TGs, on LVstructure may be related to cardiac steatosis, a recently recognized cardiometabolic condition that associates hypertriglyceridemia with LVH. In physiological conditions, most of the energy used by the myocardium is derived from beta oxidation of fatty acids. Most of the fatty acids inserting myocardium are used for energy production, whereas only a small amount is stored in the intracellular myocardial lipid pool^21,22^. When there is an imbalance between lipid storage and lipolysis in cardiomyocytes, cardiac steatosis and myocardial hypertrophy are observed^23^. Human studies have demonstrated an increased myocardial triglyceride content in patients with diabetes mellitus II^24^ and generalized lipodystrophy^25^, which was independently associated with concentric LV remodeling in these group of patients. Use of imaging modalities, such as the magnetic resonance spectroscopy, for the measurement of myocardial lipid content in hypertensive patients, may elucidate the potential role of cardiac steatosis as an underlying mechanism for the association of LVMI with plasma TGs.

The intriguing finding of the disassociation of LVMI with plasma TGs between men and women, also merits attention. A possible explanation for the lack of an independent association between LVMI and plasma TGs in women may be attributed to the difference in tissue metabolic profile and myocardial energy use between the two genders. Recently, an experimental study showed that females, compared to males, may be protected from cardiomyopathy through mechanisms related to genetically-determined normal cardiac glucose uptake and preserved cytochrome c oxidase activity^26^, a mitochondrial enzyme regulating tissue metabolism and energy production. Future, translational research studies need to confirm the above, novel, experimental data and shed new light into the pathophysiology of LVH.

Finally, the absence of measured insulin levels and the subsequent estimation of insulin resistance that could explain, at least in part, the observations of the present study, may be considered as a possible limitation of our study. Moreover, the lack of studying a non-hypertensive group should also be taken into consideration given that the demonstration of a strong association of plasma TGs with LVMI in a non-hypertensive, high risk population would strengthen the results of the present study and would extend the clinical implications in other populations.

## Conclusion

Although no etiological relationships can be established from the present study, our intriguing finding of the independent relationship of LVMI with plasma TGs in hypertensive patients may have important clinical implications. Considering the adverse prognostic role of LVH, it might be suggested that the plasma TGs, may explain part of the residual cardiovascular risk observed in patients with optimal plasma LDL-C levels. Moreover, TGs may serve as a useful marker for identifying hypertensive patients at high risk of LVH. Future studies need to clarify whether treatment of increased plasma TGs may promote LVH reduction in hypertensive patients. Finally, the gender discrepancy regarding the association of LVMI with plasma TGs provides new insights into the pathophysiology of LVH in both genders and gives the impetus for further research.

## Methods

### Study population

This is a cross-sectional study. We studied 760 non-diabetic, never treated, hypertensive patients recruited from Hypertension Unit of 1st Cardiology Department from 2007 to 2015. Arterial hypertension was defined according to the European Society of Hypertension Guidelines^27,28^. Briefly, office blood pressure (BP) was measured by an oscillometric sphygmomanometer, taking at least three measurements spaced by 1 min, allowing the patients to rest for 10 min before examination. Measurement of brachial SBP ≥ 140 mmHg and DBP ≥ 90 mmHg were considered as systolic and diastolic arterial hypertension, respectively. Mean arterial pressure was calculated as DBP + 1/3 (SBP-DBP).

Participants were subject to transthoracic echocardiography (GE ultrasound system, Vivid 3) and LVH was assessed by estimating left ventricular mass (LVM) using the Devereux formula: LVM (g) = 0.8 × 1.04 × [(LVDd + IVS + PW)^3^ – LVDd^3^] + 0.6. Left ventricular mass was divided by body surface area (BSA) to obtain left ventricular mass index (LVMI). Body surface area was calculated according to the Dubois formula (BSA = 0.007184*Height^0.725^*Weight^0.425^). Left ventricular hypertrophy was defined as LVMI > 115 g/m^2^ and > 95 g/m^2^ for males and females, respectively^27,28^.

Plasma total cholesterol, HDL-C, LDL-C, TGs and glucose were measured with standard techniques by autoanalyzers after fasting for 12 h. Patients with fasting glucose levels ≥ 100 mg/dl were subject to 2 h oral glucose tolerance test. Plasma levels of ApoA1 and ApoB were measured by nephelometry (QUED, OSAN for ApoA1 and ApoB, respectively, Behring Diagnostics, Marburg, Germany).

Patients with heart failure, significant valvular disease, cerebrovascular disease and chronic, systemic diseases were excluded from the study. Lipid lowering treatment was also an exclusion criterion. Height and weight were recorded and body mass index (BMI) was calculated. The study was conducted according to the principles of Helsinki Declaration. All patients gave their informed consent to participate in the study which was approved by the Institutional Research and Ethics Committee (Athens Medical School, University of Athens).

### Statistical analysis

Continuous variables are presented as mean value ± standard deviation or median value. Categorical variables are presented as absolute frequencies and percentages. Normal distribution of continuous variables was evaluated by the Kolmogorov–Smirnov test and graphically by histograms.

Difference in baseline characteristics between males and females were assessed by independent samples Student’s t-test or non-parametric Mann–Whitney test for continuous variables and chi-squared test for nominal ones. Multiple regression analysis was used to test the association of plasma TGs with LVMI after adjustment for age, gender, smoking, BMI, SBP, plasma fasting glucose, LDL-C and ApoB/ApoA1 ratio.

In order to assess the differential effect of gender on LVMI, we forced included relevant interaction terms (i.e. gender*parameter of interest) in the final model. Nested regression models, with and without interaction, were compared by Log-Likelihood ratio test. In case of significant interaction-term, stratified analysis by gender was performed. Finally, exploratory analysis implemented dichotomous LVMI (LVH yes/no) and a. evaluated the independent association of plasmaTGs with the outcome (LVH) in the multiple regression analysis and b. assessed the prognostic significance of plasmaTGs beyond the model that included age, gender, SBP and LDL-C by calculating continuous NRI^29^.

Exact p values < 0.05 were considered as statistically significant. Data analysis was performed with SPSS software, version 17.0 (Chicago, IL, USA).
